# Motion analysis of sun salutation using magnetometer and accelerometer

**DOI:** 10.4103/0973-6131.60046

**Published:** 2009

**Authors:** SN Omkar, Meenakshi Mour, Debarun Das

**Affiliations:** Department of Aerospace Engineering, Indian Institute of Science, Bangalore, India

**Keywords:** Accelerometers, gyroscopes, gravitational component, kinematic component, magnetometers, sun salutation

## Abstract

**Background:**

Sun salutation is a part of yoga. It consists of a sequence of postures done with synchronized breathing. The practice of few cycles of sun salutation is known to help in maintaining good health and vigor. The practice of sun salutation does not need any extra gadgets. Also it is very much aerobic and invigorates the body and the mind. sun salutation, which comprises 10 postures, involves most of the joints of the body. Understanding the transition phase during motion is a challenging task, and thus, new convenient methods need to be employed.

**Aims:**

The purpose of this study was to get an insight into the motion analysis of sun salutation during the transition from each of the 10 postures.

**Materials and Methods:**

A device MicroStrain sensor 3DM-GX1, which is a combination of magnetometers, accelerometers, and gyroscopes was used to measure the inclination and the acceleration of the body along the three axes. The acceleration obtained was then separated into gravitational and kinematic components.

**Results and Conclusions:**

The value of the gravitational component helps us to understand the position of the body and the kinematic component helps us to analyze the grace of the motion.

## INTRODUCTION

**Figure 1:**
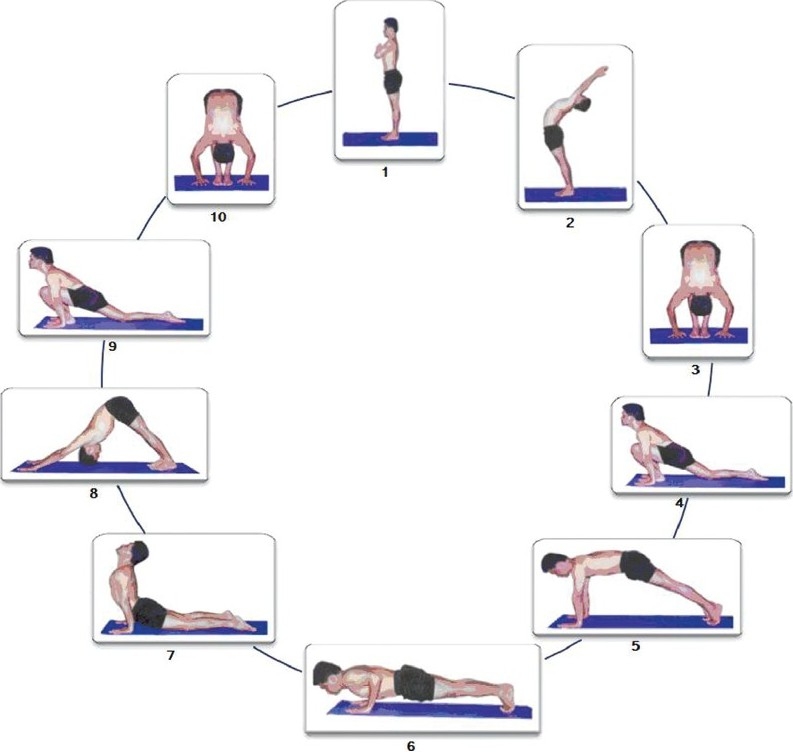
Cycle representing the 10 stages of un salutation

Many metals when combined appropriately and in right proportion yield an alloy, which has better properties. The best of orchestras is a harmonious combination of various instruments. Likewise, a sequence of postures when performed in synchronization with breathing becomes sun salutation [Figure 1]. This sequence consists of 10 subtly powerful postures set in a dynamic form performed in a single, conscious, graceful flow. The postures have been ingeniously combined with forward-bending poses countered with backward-bending ones. As the rays of the sun reaches every part of the globe, these sequence of postures ensures that the internal energy reaches every part of the body, hence the name sun salutation. Sun salutation does not need any gadgets and can be done in a limited frame of time and space. With the regular practice of the sun salutation, all the parts of the body are exercised and rejuvenated with vitality. Sun Salutation is practiced by millions of people. Different people perform it differently. In sun salutation, transition from one posture to another needs to be understood clearly to quantify the grace in the transitive pattern. The tool required to understand this transition is motion analysis.[1]

Research that has evaluated the energy expenditure of yoga indicates that yoga is essentially equivalent to moderate forms of exercise.[2] The available evidence suggests that the practice of yoga may be associated with an improvement in cardio respiratory fitness[3,4] and both muscular strength and endurance.[5] A single study that evaluated the heart rate for standing yoga postures found lower heart rate and higher rate of perceived exertion for the yoga posture sequence compared with treadmill walking.[2] All of this suggests the need for research on fitness-related outcomes associated with the practice of yoga.[6]

In the areas of medicine, sports, video surveillance, and biomechanics, human motion analysis has become an investigative and diagnostic tool. Motion analysis is done to study the kinematic and the kinetic characteristics of the human system. Human gait, defined as the pattern of human locomotion, can be described by these kinetic or kinematic characteristics.[7]

Optical motion analysis systems are often used in the study of human movement. Optokinetic measurement system and force plates may be used to evaluate to what extent subjects are able to perform certain activities of daily living in a laboratory setting.[8] However, these motion analysis systems are quite expensive, difficult to be operated with only a limited capture volume,[9] and markers are easily obscured from vision resulting in incomplete data.[10] Thus, these systems cannot measure the extent of these activities actually performed in daily living in the domestic environment, which is important for an adequate assessment of disabilities.[11] Thus, the work was initiated to obtain kinematic values using body-mounted sensors.[8,12‐17] These body-mounted sensors consisting of accelerometers and rate gyroscopes have been used to obtain kinematic values such as shank and thigh inclination angles and knee joint angle.[10,18]

Recent times have seen the progress of new technologies, powerful microcontrollers, miniature sensors, high-capacity memories, and small batteries allowing to realize low-power and portable recording systems for long-term ambulatory measurements.[19] Consequently, this has led to the developments in tracking human motion with the use of microelectromechanical accelerometers, rate gyros, and magnetometers.[20] Magnetometers are one of the few microsensors that being insensitive to acceleration can deliver absolute information about orientation in 3D space.[21] Accelerometers respond to both frequency and intensity of movement, and in this way, are superior to pedometers and actometers, which are attenuated by impact or tilt and only count body movement if a certain threshold level is passed.[22]

The study by Veltink *et al*., investigated the difference between the static and dynamic character of activities by using two or three uni-axial accelerometers mounted on different body segments. They used the accelerometer as an inclinometer based on the assumption that the magnitude of acceleration can be neglected with respect to gravity.[8] However, this method gave unacceptable errors in dynamic human motion recording.[20]

Study by Angelo M. Sabatini *et al*., showed that gyroscope signal can be used to estimate the angular velocity and can be integrated to estimate the pitch angle.[8] However, drift introduced by the integration of the angular velocity and the need for repetitive offset correction were the drawbacks of this system.[20,21] These drawbacks were overcome by R. Zhu and Z. Zhou.[20] They used Kalman filter to integrate accelerometers with rate gyros and magnetometers, and their results showed excellent dynamics of gyro and stable driftfree performance of gravity acceleration and magnetic field.

H. Dejnabadi, B. Jolles, and K. Aminian presented a new method to estimate flexion extension angles based on a combination of accelerometers and gyroscopes, which was found to be very accurate. Their model of uni-axial joint rotation could be extended to multi-axial joint rotation by employing 3D-accelerometers and 3D-gyroscopes on each site.[19]

Assessment of daily physical activity was done using tri-axial accelerometer (TA) and a portable data processing unit.[22] However, the system was found to have low sensitivity to sedentary activities and was unable to register static exercise.

S. Bonnet and R. Heliot investigated the kinematic analysis of human movement using body-mounted magnetic field sensors. Their study showed that, in dynamic situations, magnetometers provided orientation data, and using these data combined with the data provided by the accelerometer, the kinematical and gravitational component of acceleration could be separated with respect to body frame.[21]

The typical outcome of a general-purpose motion analysis describes different mechanical quantities expressed in terms of the inertial reference frame. However, noninertial frames allow equations of motion to be simplified significantly. Using noninertial frames and subsequently describing different motion quantities in such frames can enrich the analysis to a great extent. Thus, our motion analysis of sun salutation is investigated with reference to the body frame.

The purpose of our study was to estimate the trunk inclination while performing the 10 postures of sun salutation and the acceleration of the body involved in the transition from one posture to another. There are two major schools of sun salutation, one consisting of 12 steps and the other consisting of 10 steps in each round. In the mode of sun salutation having 12 steps, stage 2 is repeated in the end and stage 1 is considered twice. Since we have ignored the repetition stage 1 and 2, the mode of sun salutation used in our study comprises 10 steps.

The full cycle of sun salutation was performed by the author with the MicroStrain sensor 3DM-GX1 attached on the lower back at the center of gravity of the body. The accelerations that we measured using this instrument were relative to the initial body frame. The measured accelerations were then separated into its gravitational and kinematic component and necessary discussions followed based on the data obtained.

## MATERIALS AND METHODS

This article analyzes the acceleration of the body in the three axes while performing sun salutation based on a combination of magnetometers, accelerometers, and gyroscopes. The device used is a MicroStrain sensor 3DM-GX1, which combines three angular rate gyros with three orthogonal DC accelerometers, three orthogonal magnetometers, multiplexer,16 bit A/D converter, and embedded microcontroller to its output orientation in dynamic and static environment. The device is calibrated for sensor misalignment and gyro G-sensitivity.

The place of attachment of the device on the human body is an important issue.[23,24] Arrangements were made to make sure that the device on the human body did not cause any disturbance during sun salutation. Since the kinematic and gravitational components of the output are dependent on the measurement location, the device was attached at the trunk (second lumbar vertebra) as this segment represents the major part of total body mass.

The device gives data, such as pitch angle, roll angle, yaw angle, and acceleration along the three axes of the sensor, in tabular form for analysis. While a body is in dynamic situation, the acceleration of the body accounts for its motion. The acceleration data are obtained from a single subject undergoing five cycles. The average of this acceleration is used for the purpose of our analysis. From the data obtained, it is possible to separate these gravitational and kinematic components of acceleration. The gravitational component is dependent on the orientation of the instrument with respect to the gravitational vector of the earth. It influences the total output considerably, especially when the angle between the measurement direction and the gravitational vector of the earth is relatively large and the kinematic component of instrument output is small.

**Figure 2:**
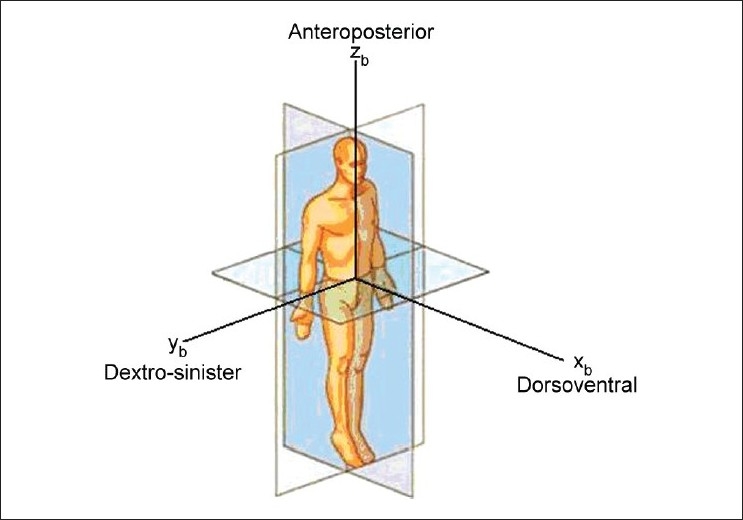
Human model showing the body axes

While studying the motion analysis of the body during sun salutation, we need to identify three axes along which the movement of the body takes place. The three axes [Figure 2] are

- The dorsoventral axis denoted by the *x_b_* axis
- The dextro-sinister axis denoted by the *y_b_* axis
- The anteroposterior axis denoted by the *z_b_* axis

**Figure 3:**
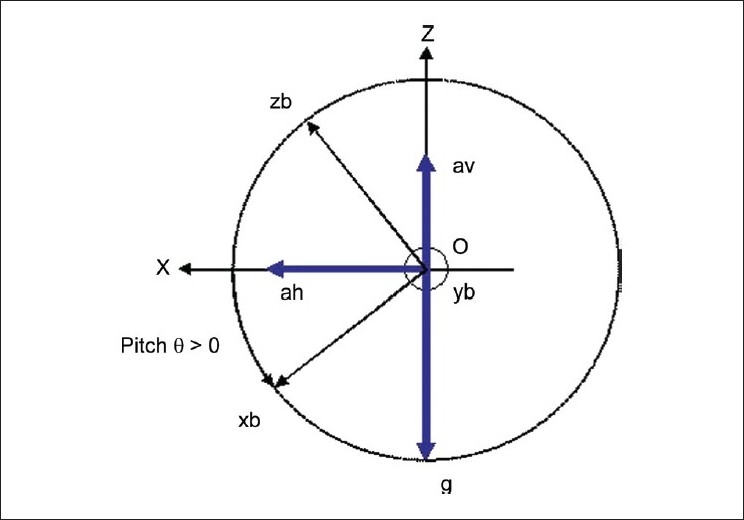
Acceleration vectors in inertial and body frame index: Z = Inertial *Z*-axis, X = Inertial *X*-axis, θ= Pitch angle

The accelerometer gives the net acceleration along the three axes of the device as shown in Figure 3. This acceleration has kinematic and gravitational component in it. The separation of the gravitational and the kinematical components is done using the following steps:

- The vector representing the gravitational component (g/B) can be expressed in sensor coordinate frame by

$$g/B=\left|\begin{array}{c} \sin\theta \;\\ 0\\ -\cos\theta \end{array}\right|$$            (1)

where, (sin *θ*) and (-cos *θ*) are the gravitational component along the *x_b_* and *z_b_* axes, respectively.

- The acceleration vectors can be expressed in the inertial and the body frame [Figure 3].

- The values of *α_h_* and *α_v_* are computed as shown in equations (2) and (3).[21]

$$\alpha_{h}≃-\alpha_{\mathrm{xb}}\cos\theta -v_{\mathrm{zb}}\sin\theta$$            (2)

$$\alpha_{v}≃\alpha_{\mathrm{xb}}\sin\theta -v_{\mathrm{zb}}\cos\theta +1$$            (3)

where *α_h_* is the horizontal acceleration along the U-axis

*α_v_* is the vertical acceleration along the Z-axis

*α_xb_* is the sensor readings along the *x_b_* axis

*α_zb_* is the sensor readings along the *z_b_* axis

- Using formulas (2) and (3), we compute the kinematic component of acceleration based on our observation of Figure 3.

- Kinematic component along *z_b_* axis

$$\alpha_{\mathrm{zb}}=(\alpha_{v}\;\cos\theta )\;+\;(\alpha_{h}\;\sin\theta )$$           (4)

- Kinematic component along *x_b_* axis

$$\alpha_{\mathrm{xb}}=(\alpha_{h}\;\cos\theta )\;-\;(\alpha_{v}\;\sin\theta )$$           (5)

## RESULTS AND DISCUSSIONS

**Figure 4:**
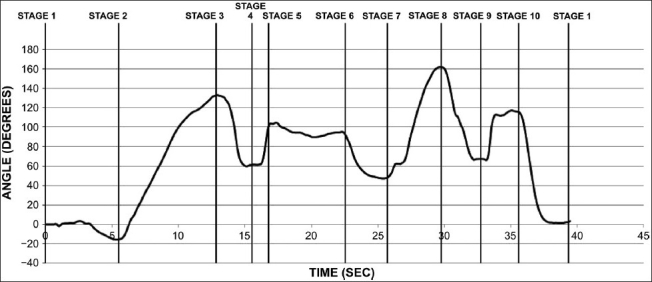
Pitch angle variation of the body for one full cycle of sun salutation

During sun salutation, the movement of the body is along the dorsoventral axis denoted by the *x_b_* axis. This can be clearly seen by the plotting the graph of the measured pitch angle for the full cycle [Figure 4].

In the following graph, the body undergoes maximum pitching about 100°C while executing posture 3 from posture 2. Notably, a significant amount of pitching can be observed during stages 7, 8, and 10.

**Figure 5:**
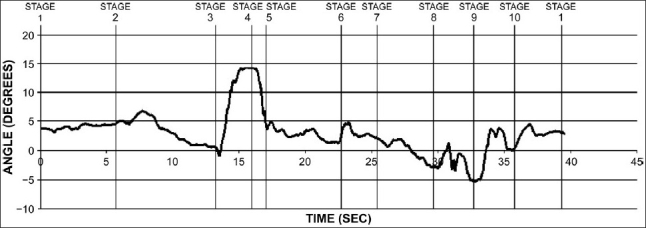
Roll angle variation of the body for one full cycle of sun salutation

While performing these postures, the body also undergoes some amount of rolling. To perform a graceful sun salutation, there must be minimal amount of this ungainly encumbrance. Beginners who are stiff tend to have more rolling. The lesser the roll angle more will be the grace. Hence, measuring the roll angle during sun salutation is of great help assessing the performance of the practitioner [Figure 5].

**Figure 6:**
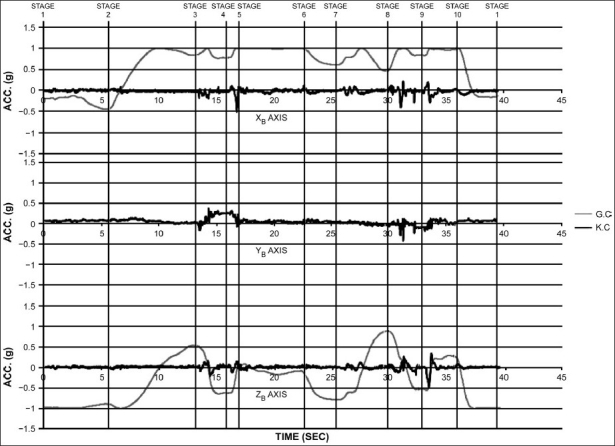
Separation of the gravitational and kinematical components. Accelerations during a cycle of sun salutation expressed in the sensor frame

The variation of roll angle is minimal except for stage 4 and 9, where the subject brings one leg forward, and in order to do that, the body has to undergo some amount of rolling movement. The kinematic and gravitational components of acceleration resolved using equations (1) to (5) are plotted [Figure 6] for the motion analysis from posture to posture transition.

In Stage 1, the subject stands upright, and hence, all the component of acceleration due to gravitation is seen along the *z_b_* axis (which coincides with the inertial *Z*-axis) with no component along the *x_b_* axis.

In Stage 2, the subject bends the body backwards, and as a result, we see that there is a gravitational component along the *x_b_* axis in the negative direction. And there is a slight decrease in the gravitational component along *z_b_* axis.

During the transition phase from Stage 2 toStage 3, there is an instance when the trunk becomes parallel to the ground. At this instance, the *z_b_* axis becomes perpendicular to the gravitational component and is aligned along the inertial X axis momentarily. This is reflected in the null value of gravitational component along the *z_b_* axis. Consequently, the *x_b_* axis aligns itself along the inertial Z axis, and hence, the entire acceleration due to gravity is seen along this axis. Upon the completion of stage 3 where the subject bends down completely, the instrument is in an inclined position reflecting gravitational components in both *x_b_* and *z_b_* direction.

While executing Stage 4, again the trunk becomes horizontal momentarily and then is inclined at an angle resulting in the gravitational components of acceleration in both *x_b_* and *z_b_* direction.

In Stages 5 and 6, the trunk is parallel to the ground and there is minimal deviation of the trunk angle as the body transits from stage 5 to 6. This is reflected by a straight line on the graph.

During Stage 7, the body takes the form of an arc. Here, again the magnetometer is at an inclination similar to that in stage 4.

While proceeding towards Stage 8, the momentary horizontal inclination of the trunk is again seen. Upon the execution of this stage, we see a distinct component of gravitational acceleration on the two axes due to the acute inclination of the trunk.

The concluding stages of sun salutation (Stages 9 and 10) are merely repetitions of stages 4 and 3, respectively.

During the execution of sun salutation, the component of acceleration along the *y_b_* axis is almost negligible except in stages 4 and 9. In these stages, the subject tries to bring one leg forward, which involves a rolling movement of the body. It is this rolling that gives rise to a kinematic component.

The duration from stage 1 to stage 2 and stage 2 to stage 3 is nearly equal, whereas stages 3 to 4 and 4 to 5 are performed in a rapid transition. Stages 5 to 10 are performed in almost a uniform manner though the duration is a bit more from stage 5 to stage 6.

The primary purpose of our study was to investigate posture to posture transition during sun salutation. The nature of the transition, of being smooth or rough, is determined by the variation of the kinematic component of acceleration. Figure 6 clearly indicates peaks in the kinematic component along all the three axes from stage 3 to 5 and from stage 8 to 9, which reflects a rough transition while executing these postures.

This article extends the work done on motion analysis using inclinometer and uses it to analyze the motion of the body while performing sun salutation. The value of gravitational component of acceleration varies as the body is transitioning from one posture to another and hence helps us to understand the position of the body with respect to the inertial axes. On the other hand, the value of kinematic component of acceleration depends on the uniformity of the motion and hence helps us to analyze the grace.
